# Lipidomic Modulation in Stressed Albino Rats Is Altered by Yolk and Albumen of Quail (*Coturnix japonica*) Egg and Poultry Feed

**DOI:** 10.1155/2016/2565178

**Published:** 2016-01-28

**Authors:** Emmanuel Oluwafemi Ibukun, Gideon Oludare Oladipo

**Affiliations:** Department of Biochemistry, Federal University of Technology, Akure, Ondo State, Nigeria

## Abstract

Cold and immobilization stressors can generate oxidative stress as well as skeletal muscle fatigue. Free radicals cause oxidative degradation of lipids, proteins, nucleic acids, and carbohydrates molecules, thereby compromising cell integrity and function. Quail egg had been described as being very functional biochemically, due to the essential biomolecules it contains in very regulated quantity. This study was aimed for evaluating the dietary effect of the egg on lipid profile parameters on selected tissues. The antilipidemic properties of the egg yolk and albumen and poultry (layers) feed were determined in selected tissues in male albino rats assaulted with cold immobilization stress induced on them at 4°C for 2 hours, while diazepam was used as standard antistress drug. Antilipidemic activities were evaluated by lipid profile modulation (HDL, LDL, TRIG., and T-CHOL.). Quantitative and qualitative analyses of fatty acids profile of the yolk hexane-extract were determined by Gas Chromatography and Mass Spectrophotometry (GC-MS). The ameliorative impacts of diazepam (2.5 and 5.0 mg/mL/kg BW), yolk (5 and 10 mL/kg BW), albumen (5 and 10 mL/kg BW), and the feed (5–10 mg/kg BW) were competitively (*p* < 0.05) specific for each of the tissues. The result of the study suggested yolk and albumen of quail egg and poultry feed as antistress agents as well as lipid modulators.

## 1. Introduction

Stress is considered to be the condition which results in perturbation of the body's homeostasis [1]. If the level of stress is extreme, the homeostatic mechanisms of the organism become deficit and the survival of the organism is threatened [2]. Stress has been postulated to be involved in the etiopathogenesis of a variety of disease states, namely, hypertension, peptic ulcer, diabetes, immunosuppression, reproductive dysfunctions, and behavioural disorders like anxiety due to involvement of the central nervous system (CNS), endocrine system, and metabolic system [3]. Drugs having antistress properties induce a state of nonspecific resistance against stressful conditions [4]. Drugs like benzodiazepines, certain CNS stimulants such as amphetamines and caffeine, and some anabolic steroids are routinely used to combat stress [4]. The incidence of toxicity and dependence has limited the therapeutic usefulness of these drugs [5, 6].

The key components of the “stress system” are the hypothalamic-pituitary-adrenal (HPA) axis and the sympathetic nervous system (SNS) [7]. When the hypothalamus is triggered by a stressor, corticotropin-releasing hormone (CRH or CRF, corticotropin-releasing factor) and arginine vasopressin (AVP) are secreted, eliciting both the production of adrenocorticotropin hormone (ACTH) from the posterior pituitary and the activation of the noradrenergic neurons of the locus coeruleus/norepinephrine (LC/NE) system in the brain [7]. Under normal conditions, the production of CRH and ACTH fluctuates in a predictable circadian cycle and they are inhibited by high levels of blood cortisol via a well-described negative feedback loop [7]. Experimental and clinical evaluations are specific for a wide range of body changes also called adaptation syndrome, which are predictable rhythm and responses of the HPA axis [7].

Also stress has been reported to elevate the level of total cholesterol in the body and total cholesterol has been declared a culprit behind various life threatening diseases like hypertension, cardiovascular diseases, atherosclerosis, obesity, hypercholesterolemia, metabolic syndrome, and even diabetes [8–10]. Due to its hydrophobic nature, cholesterol is transported by lipoproteins, and various types of these lipoproteins have been identified with the two most abundant known as low density lipoprotein (LDL) and high density lipoprotein (HDL) [8]. Hypercholesterolemia resulting from stressors is usually characterized by both abnormal serum and hepatic triglyceride and cholesterol levels [11]. Increased serum total cholesterol may have caused impairment in the triglyceride metabolism leading to the accumulation/deposition of free fatty acids in the liver, thus triggering a condition known as fatty liver [11]. This expanded liver fatty acid pool leads to increased mitochondrial and peroxisomal *β*-oxidation, which produces reactive oxygen species. This may, in turn, promote a local proinflammatory state leading to progressive liver injury [12].

Cardiovascular disease (CVD) is one of the most common diseases in the Western world; its complex aetiology involves both genetic and acquired factors. Public health messages about diet and CVD have tended to focus on saturated fatty acids (SFA) and n-6 PUFA [13]. More recently, the messages have included the positive role of n-3 PUFA [13]. It had been suggested in animal and human studies that n-3 PUFAs have an immunomodulatory effect [14].

Blood lipids are influenced by nutrition, body weight, physical activity, medications, and genetic factors [15, 16]. Evidence suggests that blood lipids are also affected by mental status [17]. It is postulated that stress increases blood lipids through increasing hepatic lipoprotein lipase activity caused by a heightened sympathetic neuronal response [18]. Study demonstrated by Fakhari et al. (2007) revealed an increase in serum triglycerides in individuals exposed to stress in the past 6–12 months [19]. Few studies have investigated a possible link between high density lipoprotein cholesterol (HLD-C) and low density lipoprotein cholesterol (LDL-C) levels and stress [20].

Cold immobilization stress also called immobilization stress and cold restraint stress had been described by Popovic et al. (2009) as experimental induction of very extreme condition which cannot be carried out on human but is believed to have same effect on human as it is revealed in animal models [21].

This study was aimed at evaluating the antistress effects of quail egg yolk and albumen and poultry feed on the lipid modulations in selected tissues and determining the toxicological evaluation of diazepam used as standard antistress drug on lipid modulation. The poultry feed antistress activity was evaluated to predict its possible contribution to the antistress properties of the egg components, since it had been predicted that some bioactive supplements of feed were found to improve the antioxidant status of the egg yolk of quail [22]. The profiling of fatty acids in the lipid extracts of the yolk was determined.

## 2. Materials and Methods

### 2.1. Materials

#### 2.1.1. Reagents and Chemicals

Reagents and chemicals used in this experiment were obtained from different sources such as British Drug House (BDH) and were all of good analytical grades and analytical kits were purchased from local distributors of Randox Laboratories Limited. All the solutions, buffers, and reagents were prepared using glass distilled water. Diazepam was used in this experiment as standard antistress drug (reference drug) and obtained from Martadol Pharmaceutical Shop, Akure, Ondo State, Nigeria, and was NAFDAC registered.

#### 2.1.2. Sample Collection

The quail egg samples were collected by rearing of a mature female* C. japonica* being fed with the poultry layers' feed. Eggs were carefully handled in an icebox. They were kept in the refrigerator at 4°C before the analyses. Yolk and albumen of egg and poultry feed were collected and constituted in distilled water to make concentrations of 5 and 10 mg/mL.

#### 2.1.3. Lipid Extraction

Lipid from the quail egg yolk was extracted using AOAC (1990) with little modification. 150 g of yolk was dissolved in chloroform (300 mL) in a test tube fitted with a condenser, and 1% sulfuric acid in methanol (150 mL) was added, mixture was vortexed for 2 hours and 50 mL of sodium chloride solution (5% w/v) was added, and the required esters were extracted with 50 mL of hexane. Pasteur pipettes were used to separate the layers. The hexane layer was washed with 50 mL of 2% w/v potassium bicarbonate solution and dried over anhydrous sodium sulfate. The solution was harvested to remove the drying agent, and the solvent was removed under reduced pressure in a rotary film evaporator. The lipid extract was then stored in an air-tight container in a desiccator at 4°C until needed. Quail egg yolk lipid extract was further methylated using standard laboratory protocol. 50 mg of the extracted fat content of the yolk was saponified (esterified) for five (5) minutes at 95°C with 3.4 mL of the 0.5 M KOH in dry methanol. The mixture was neutralized by using 0.7 M HCl. 3 mL of the 14% boron trifluoride in methanol was added. The mixture was heated for 5 minutes at the temperature of 90°C to achieve complete methylation process. The fatty acid methyl esters were thrice extracted from the mixture with redistilled n-hexane. The lipid extract was used to profile the fatty acids present in the sample using Gas Chromatography and Mass Spectrophotometry.

#### 2.1.4. Gas Chromatography and Mass Spectrophotometry

The content was concentrated to 1 mL for gas chromatography analysis and 1 *μ*L was injected into the injection port of GC. The GC equipment used was HP 6890 powered with HP chemstation Rev. A09.01 (1206) software. The split ratio was 20 : 1, and the carrier gas was nitrogen at inlet temperature of 250°C with a column type of HP INNOWax and column dimensions of 30 m × 0.25 mm × 0.25 *μ*m. The oven program parameters include initial temperature at 60°C, first ramping at 12°C/minute for 20 minutes, maintained for 2 minutes, and second ramping at 15°C/min for 3 minutes, maintained for 8 minutes. The detector used was FID at 320°C at hydrogen pressure of 22 psi and compressed air of 35 psi.

### 2.2. Experimental Design

#### 2.2.1. Immobilization Stress (IS) Combined with Cold Restraint Stress (CRS)

Male albino rats were used according to the standard guidelines of the Care and Use of Experimental Animal Resources. 50 male albino rats weighing 200 ± 10 g were used to evaluate the ability of the quail egg yolk and albumen as well as feed to combat stress and were obtained from standard animal house. The rats were housed 5 per cage under constant environmental conditions (20–24°C; 12 h light/dark cycle) and were given ad libitum access to standard pelleted food and water. After the administration of quail egg yolk and albumen and poultry feed for 21 days combined IS and CRS test was performed by immobilizing animals in the cold chamber at 4 ± 0.3°C; the plexiglass cage volume was adjusted to the size of the animal, to restrain completely their movements [21] for 2 hours, except group 1. Group 2 animals were treated after stress with diazepam (5 and 10 mg/mL/kg BW). Immobilization was done in a plastic container with the aid of paper glue:

 Group 1: untreated, unstressed group-negative control group (C−).
 Group 2: untreated, stressed group-positive control group (C+).
 Group 3: diazepam treated (5.0 mg/mL/kg/BW), stressed group (D1).
 Group 4: diazepam treated (2.5 mg/mL/kg/BW), stressed group (D2).
 Group 5: treated (albumen 5 mg/mL/kg/BW), stressed group (A1).
 Group 6: treated (albumen 10 mg/mL/kg/BW), stressed group (A2).
 Group 7: treated (yolk 5 mg/mL/kg/BW), stressed group (Y1).
 Group 8: treated (yolk 10 mg/mL/kg/BW), stressed group (Y2).
 Group 9: treated (feed 5 mg/mL/kg/BW), stressed group (F1).
 Group 10: treated (feed 10 mg/mL/kg/BW), stressed group (F2).

#### 2.2.2. Preparation of Serum and Tissue Homogenates

The rats were sacrificed by cervical dislocation. Blood samples were collected by ocular punctures into plain bottles. Serum was prepared by aspiration of the clear yellowish liquid after clotting and centrifuged for 10 minutes at 3000 g in a bench centrifuge. The clear supernatant was used for the estimation of serum lipids. The animals were quickly dissected and the lung, brain, heart, liver, and kidneys removed and rinsed with ice-cold 1.15% potassium chloride. The tissues were then homogenized in ice-cold 0.1 M phosphate buffer solution using a Teflon homogenizer.

### 2.3. Biochemical Estimation

#### 2.3.1. Determination of Triglyceride Concentration

The triglyceride concentration was determined using colorimetric method as described by Tietz (1982) [23] as described in the kit's manufacturer (Randox Laboratories Ltd.) manual. Briefly, 10 *μ*L of the sample was mixed with 1 mL of Pipes reagent (40 mM phosphate buffer, 5.5 mM 4-chlorophenol, and 17.5 mM Mg^2+^) and enzyme reagent (4-aminophenazone, adenosine triphosphate, lipase, glycerol kinase, glycerol-3-phosphate oxidase, and peroxidase). Thereafter the mixture was incubated for 5 min at 37°C and the absorbance at 546 nm was taken against reagent blank within 60 min using visible spectrophotometer 721(D) (UK). The triglyceride concentration was subsequently calculated against the standard.

#### 2.3.2. Determination of Total Cholesterol Concentration

The total cholesterol concentration was determined using colorimetric method by Allain et al. (1974) [24] as described in the kit's manufacturer (Randox Laboratories Ltd.) manual. 1 mL of the reacting mixture containing 4-aminoantipyrine, phenol, peroxidase, cholesterol esterase, cholesterol oxidase, and 80 mM Pipes buffer pH 6.8 was mixed with 10 *μ*L of plasma and incubated for 5 min at 37°C. The absorbance at 546 nm was then taken against the reagent blank within 60 min using visible spectrophotometer 721(D) (UK). The concentration of cholesterol in the sample was subsequently calculated against a standard.

#### 2.3.3. Determination of HDL Cholesterol Concentration

The precipitation was carried out according to the method of Lopes Virella et al. (1977) [25] as described in the kit's manufacturer (Randox Laboratories Ltd.) manual. Briefly, 200 *μ*L of plasma was mixed with 500 *μ*L of the precipitant (0.55 mM phosphotungstic acid and 25 mM magnesium chloride) and allowed to sit for 10 min at room temperature. Then, the mixture was centrifuged for 10 min at 800 ×g. Thereafter, the clear supernatant was separated off and subjected to the same procedure for the determination of total cholesterol described above.

#### 2.3.4. Determination of Plasma LDL Cholesterol Concentration

The LDL cholesterol concentration of the plasma samples was determined according to method by Friedewald et al. (1972) [26]:

(1) LDL Cholesterol mg/dL=Total Cholesterol−Triglycerides5−HDL Cholesterol.

All values are expressed as mean, standard deviation denoted by error bars. Statistical evaluation was done using One Way Analysis of Variance (ANOVA) followed by Duncan's Multiple Range Test (DMRT). The significance level was set at *p* < 0.05.

## 3. Result and Discussion

### 3.1. Fatty Acid Methyl Ester Analyses of Quail Egg Yolk Extract Using GC-MS

It is supposed that n-3 and n-6 polyenic fatty acids are metabolized by identical enzymatic systems [27]. On the other hand, none of the representatives of these two families may be transformed into an acid from another family [27]. At high concentrations of linoleic acid, conversion of *α*-linolenic acid to eicosapentaenoic and docosahexaenoic acids is inhibited. Most probably, the high proportion of linoleic acid to linolenic acid in organism negatively influences the deficiency of long-chain fatty acids [28]. The rationale for the proposed ratios relates to the belief that the two classes of fatty acids interact within the human body following parallel pathways continually competing with each other for chemical conversion to various structures without and within the cell. A dietary ratio of 4 : 1 produces almost a 1 : 1 ratio of Polyunsaturated Fatty Acids (PUFAs) in cell membranes [29, 30], while one estimate is that, in developed countries, the ratio of n-6s to n-3s is closer to 15 : 1 [31], which could be the reason for elevated CVD reports in those regions.

The qualitative and quantitative estimation of fatty acids in the quail egg yolk lipid extract revealed the fatty acids using GC-MS (Figure 5): hexadecanoic acid (c16:0), palmitoleic acid (c16:1), stearic acid (c18:0), oleic acid (c18:1), linoleic acid (c18:2), linolenic acid (c18:3), arachidonic acid (c20:4), docosahexaenoic acid (c22:6), and eicosapentaenoic acid (c20:5).

The lipid contained omega-3 fatty acid (PUFA) precursor, *α*-linolenic acid (0.82 ± 0.023), and linoleic acid (7.73 ± 1.078) which is the precursor of omega-6 PUFA. The consequence was elevated arachidonic acid (1.2 ± 0.089). Quail egg yolk had omega-6 : omega-3 PUFA ratio of 2.27.

The result revealed stearic acid of 10.04 g per 100 g of oil extracts. Stearic or octadecanoic acid, classified as saturated fatty acid (SFA), had been shown to have recommendable effects on blood total and low density lipoprotein (LDL) cholesterol levels [32]. Stearic acid's effect on blood total and LDL-C cholesterol levels indicates that this long-chain saturated fatty acid may deplete cardiomyopathy [33]. However, the effects of stearic acid on HDL-C and triglyceride levels were inconsistent [32, 33]. Palmitoleic acid is a monounsaturated fatty acid resembling saturated fatty acids in its ability to lower LDL-C [34]. The result also revealed palmitoleic acid (8.21 ± 1.021). Oleic acid is a predominant amount of 36.27 g per 100 g of lipid extract. It had been reported as LDL-C lowering fatty acid [34]. Hexadecanoic (palmitic) acid which occurred in high amount of 18.67 g per 100 g of oil extract was reported to reduce LDL as linoleic acid increases [35]. These fatty acids could be responsible for the LDL-C and total cholesterol reducing potential of quail egg yolk. The dietary effects of lipid modulation on liver, kidney, heart, brain, blood, and lung were evaluated.

### 3.2. Lipid Modulation Evaluations

The poultry feed was included in the experimental design to ascertain the contribution of the feed to the modulating activities of the quail egg yolk as well as albumen. Over the past few years, many research studies have demonstrated that feed bioactive supplements may be transferred from hens' feed into the yolk. Lutein and zeaxanthin are the most extensively studied phytochemicals in egg yolk [36]. Sahin et al. (2010) revealed that resveratrol used as feed supplements elevated the antioxidant status of the quail egg yolk [22]. It has been reported that dietary sinapic acid (4-hydroxy-3,5-dimethoxycinnamic acid) affects egg quality characteristics and low levels of sinapic acid were detected in egg yolk [37]; it was also reported that the deposition of simple phenolic acids, unlike the isoflavonoids, in egg yolk is very low under natural conditions. However, it is not known if other phenolic compounds are present in egg yolk [38]. The quail birds were given feed formulated for layers bird during the period of this research study. Although this study did not demonstrate the quality of the egg if given other feed formulations, it is expedient to understand the contribution of layers formulation to the lipid modulation with regard to stress management. The layers feed formulation was reported to contain protein (nitrogen × 6.25) (18.00%), lysine (0.94%), methionine + cysteine (0.71%), calcium (3.80%), phosphorus (0.45%), sodium (0.18%), potassium (0.68%), chlorine (0.19%), and metabolizable energy kcal/Ib (1320) [39]. These constituents were formulated in yellow corn and soybean meals. Through dietary manipulation, certain phytochemicals with important health benefits can be enriched in egg yolk.

**Figure 1:**
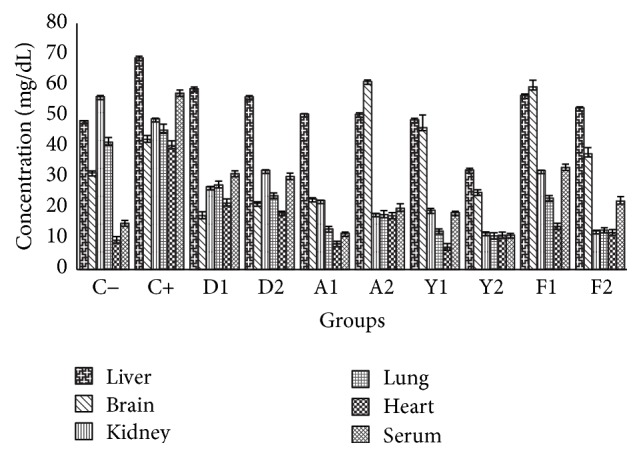
Effects of the extracts of yolk, albumen, and feed on triglyceride profiles in liver, brain, kidney, lung, heart, and serum (mg/dL). Each value represents mean (*n* = 5).

There were mark increases in triglycerides and total cholesterol levels for all the experimented tissues in the untreated stressed group (C+) of animals when compared to the unstressed and untreated group (C−) as shown in Figures 1 and 3, respectively. This established that cold immobilization stress could cause hypertriglyceridemia. The mark increase is probably due to stimulation of hypothalamus-pituitary axis (HPA) and sympathetic system, resulting in liberation of catecholamines and glucocorticosteroids, which inhibits the immune system at multiple sites including liver and kidney [40]. The advent of stress in the biosystem is envisaged to release corticosterone that is synthesized in response to adrenocorticotrophic hormone, stimulating the circulation of high-energy compounds such as glucose, free amino acids, and free fatty acids, initiating cellular proliferation. Quail egg yolk and albumen, poultry feed, and standard drug (diazepam) significantly (*p* < 0.05) reduced the elevated triglycerides and cholesterol levels as revealed in groups for all the tissues. There was a consistent high value for the liver as revealed in the total cholesterol and triglycerides profiles, and this is due to the systemic transport of triglycerides from the organs to the liver for fatty acids metabolism. The fatty acid content of the yolk (as revealed by the lipid profile analyses of the yolk) could be responsible for its anticholesterolemic activity; also, the albumen and feed showed closely similar activity. The relationship between stress and cholesterol as revealed in these results for total cholesterol, LDL-C, and HDL-C was similar to clinical study by Shahnam et al. (2010), which reported that stress levels in Iran were responsible for the increase in total cholesterol and LDL-C but reduced HDL [20]. These effects were due to inhibition of stimulation of sympathetic nervous system. It was noteworthy that the pool of fatty acids, amino acids, and other bioactive compounds present in the yolk and albumen of quail egg and poultry feed were indeed anticholesterolemic.

**Figure 2:**
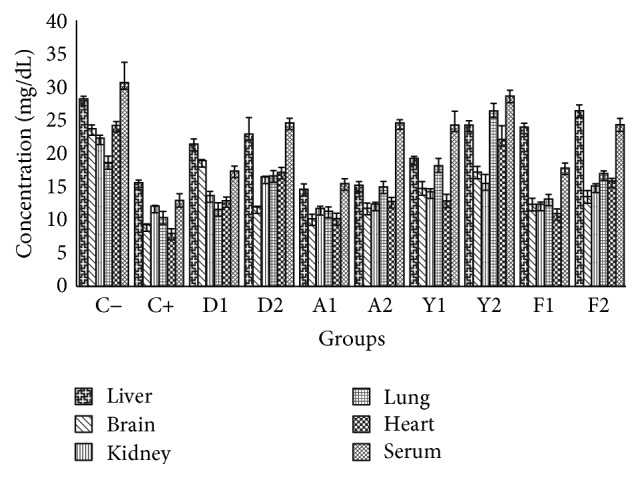
Effects of the extracts of yolk, albumen, and feed on high density lipoprotein profiles in liver, brain, kidney, lung, heart, and serum (mg/dL). Each value represents mean (*n* = 5).

**Figure 3:**
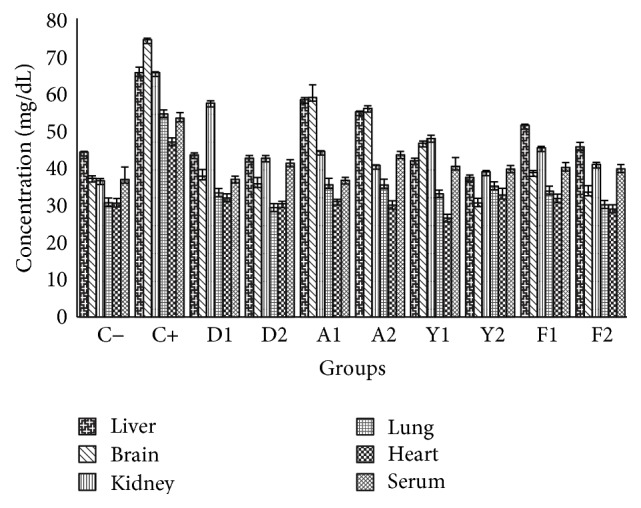
Effects of the extracts of yolk, albumen, and feed on total cholesterol profiles in liver, brain, kidney, lung, heart, and serum (mg/dL). Each value represents mean (*n* = 5).

There was an inverse relationship between the stress hormone corticosterone and HDL-C; it could be that corticosterone affected peripheral cholesterol metabolism to alter HDL cholesterol formation. The result (Figure 2) revealed that stressful condition had degenerative effect and impaired the level of HDL cholesterol as shown in the untreated and stressed group (C+) compared to the group without stress (C−); this was in agreement with Shahnam et al. (2010) [20]. However, all samples demonstrated HDL-C elevating ability.

**Figure 4:**
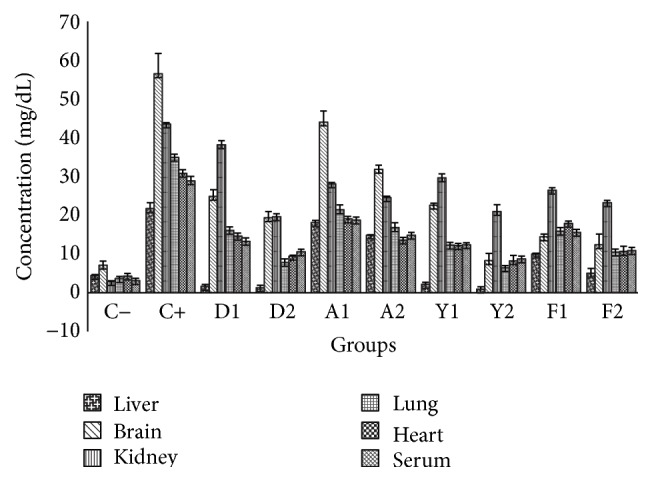
Effects of the extracts of yolk, albumen, and feed on low density lipoprotein profiles in liver, brain, kidney, lung, heart, and serum (mg/dL). Each value represents mean (*n* = 5).

**Figure 5:**
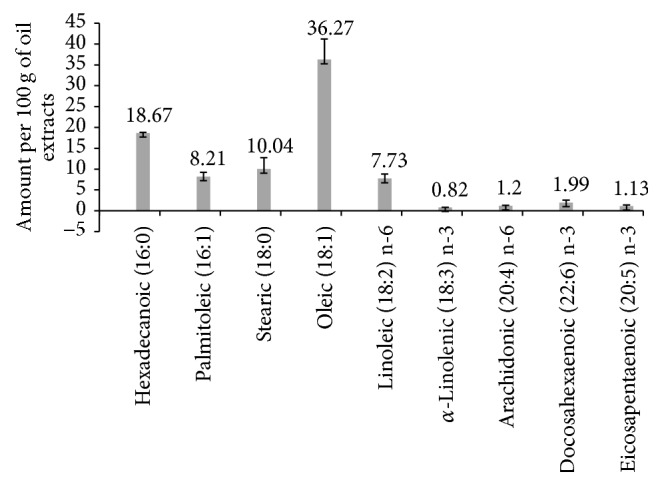
Lipid profile of* Coturnix japonica* egg yolk lipid extract. Ratio of n-6 : n-3 is 2.27.

The accumulation of LDL-C was an indication of the development of atherosclerosis.  Figure 4 revealed the LDL-C concentration. It could be observed that there was a marked increase in LDL-C levels in the tissues of the untreated stressed group (C+) when compared to the untreated unstressed (C−) and treated stressed groups. Values were significantly lower in all the tissues than the untreated stressed group. However, by significantly reducing the LDL-C level, it enhances the HDL : LDL cholesterol ratio (though not shown) and by implication the quail egg components could attenuate diseases associated with total cholesterol and LDL-C. A raised HDL : LDL cholesterol ratio was associated with low risk of diseased events. The ability of quail egg yolk and albumen extracts to significantly reduce the elevated LDL-C could be due to the antistress properties of the egg components or their antidyslipidemic activities.

The poultry feed demonstrated antistress activity that was significantly competitive with the quail egg yolk and albumen. The yolk had overall stress attenuating ability compared to the feed. Thus, it could be established that the lipid modulating potentials of quail egg yolk and albumen were essentially by natural bioactive compounds present therein. However, the contribution of poultry feed supplements to the antioxidant status of the egg could not be ignored; it could have elevated the antistress as well as lipid modulating tendencies of the egg yolk and albumen.

## 4. Conclusion

Quail egg yolk and albumen as well as poultry feed demonstrated antistress potential by ameliorating the effect of the stressor generated on lipid modulation and distribution in the tissues experimented. The study also revealed that increased stress levels were characterized by low HDL-C, high LDL-C, high total cholesterol, and high triglycerides. This validates the traditional use of quail egg in the management of degenerative diseases in relation to dyslipidaemia.

## References

[1] Khan N. M., Suresh J., Yadav K. S. H., Ahuja J. (2012). Formulation and evaluation of antistress polyherbal capsule. *Der Pharmacia Sinica*.

[2] Lakshmi B. V. S., Sudhakar M. (2009). Screening of *Psidium guajava* leaf extracts for antistress activity in different experimental animal models. *Pharmacognosy Research*.

[3] Rai D., Bhatia G., Sen T., Palit G. (2003). Anti-stress effects of *Ginkgo biloba* and *Panax ginseng*: a comparative study. *Journal of Pharmacological Sciences*.

[4] Suhail M., Kumar S. S., Sharma B. (2013). Phytoremedial role of *Withania somnifera* extract on biochemical parameters in liver of epinephrine induced stressed albino mice. *International Journal of Pharma and Bio Sciences*.

[5] Umukoro S., Ashorobi R. B. (2005). Anti-stress potential of aqueous seed extract of *Aframomum melegueta*. *African Journal of Biomedical Research*.

[6] Larsson K., Grönneberg R., Hjemdahl P. (1985). Bronchodilatation and inhibition of allergen-induced bronchoconstriction by circulating epinephrine in asthmatic subjects. *The Journal of Allergy and Clinical Immunology*.

[7] Thomas G. G., Lena E. (2010). Chronic stress and the hpa axis: clinical assessment and therapeutic considerations. *A Review of Natural and Nutraceutical Therapies for Clinical Practice*.

[8] Nelson L. D., Cox M. M., Freeman W. H. (2008). Protein function. *Lehninger Principles of Biochemistry*.

[9] Colpo A. (2005). LDL cholesterol: bad cholesterol, or bad science?. *Journal of American Physicians and Surgeons*.

[10] Murray R. K., Granner D. K., Mayes P. A., Rodwell V. W. (2003). *Harper's Illustrated Biochemistry*.

[11] Wang Y.-M., Zhang B., Xue Y. (2010). The mechanism of dietary cholesterol effects on lipids metabolism in rats. *Lipids in Health and Disease*.

[12] Schwimmer J. B., Pardee P. E., Lavine J. E., Blumkin A. K., Cook S. (2008). Cardiovascular risk factors and the metabolic syndrome in pediatric nonalcoholic fatty liver disease. *Circulation*.

[13] Ruxton C. H. S., Reed S. C., Simpson M. J. A., Millington K. J. (2004). The health benefits of omega-3 polyunsaturated fatty acids: a review of the evidence. *Journal of Human Nutrition and Dietetics*.

[14] Calder P. C. (1996). Immunomodulatory and anti-inflammatory effects of n-3 polyunsaturated fatty acids. *Proceedings of the Nutrition Society*.

[15] The Expert Panel (1998). Report of the national cholesterol education program expert panel on detection, evaluation, and treatment of high blood cholesterol in adults. *Archives of Internal Medicine*.

[16] Hunt S. C., Hasstedt S. J., Kuida H., Stults B. M., Hopkins P. N., Williams R. R. (1989). Genetic heritability and common environmental components of resting and stressed blood pressures, lipids, and body mass index in utah pedigrees and twins. *American Journal of Epidemiology*.

[17] Patterson S. M., Gottdiener J. S., Hecht G., Vargot S., Krantz D. S. (1993). Effects of acute mental stress on serum lipids: mediating effects of plasma volume. *Psychosomatic Medicine*.

[18] Bachen E. A., Muldoon M. F., Matthews K. A., Manuck S. B. (2002). Effects of hemoconcentration and sympathetic activation on serum lipid responses to brief mental stress. *Psychosomatic Medicine*.

[19] Fakhari A., Ebrahimzadeh M., Shiva S., Fekrat S., Mohammadpoorasl A. (2007). Effect of mental stress on serum triglyceride level. *Research Journal of Biological Sciences*.

[20] Shahnam M., Roohafza H., Sadeghi M., Bahonar A., Sarrafzadegan N. (2010). The correlation between lipid profile and stress levels in Central iran: isfahan healthy heart program. *ARYA Atherosclerosis Journal*.

[21] Popovic M., Janicijevic-Hudomal S., Kaurinovic B., Rasic J., Trivic S., Vojnović M. (2009). Antioxidant effects of some drugs on immobilization stress combined with cold restraint stress. *Molecules*.

[22] Sahin K., Akdemir F., Orhan C., Tuzcu M., Hayirli A., Sahin N. (2010). Effects of dietary resveratrol supplementation on egg production and antioxidant status. *Poultry Science*.

[23] Tietz N. W. (1982). *Fundamentals of Clinical Chemistry*.

[24] Allain C. C., Poon L. S., Chan C. S. G. (1974). Enzymatic determination of total serum cholesterol. *Clinical Chemistry*.

[25] Lopes Virella M. F., Stone P., Ellis S., Colwell J. A. (1977). Cholesterol determination in high density lipoproteins separated by three different methods. *Clinical Chemistry*.

[26] Friedewald W. T., Levy R. I., Fredrickson D. S. (1972). Estimation of the concentration of low-density lipoprotein cholesterol in plasma, without use of the preparative ultracentrifuge. *Clinical Chemistry*.

[27] Małgorzata K., Bogdan J., Małgorzata K., Tadeusz T., Zbigniew D. (2005). Comparative analysis of fatty acid profile and cholesterol content of egg yolks of different bird species. *Polish Journal of Food and Nutrition Sciences*.

[28] Bartnikowska E., Obiedzinski M. (1997). Unsaturated fatty acids omega-3. I. Structure, sources, determination, metabolism in the organism. *Roczniki Państwowego Zakładu Higieny*.

[29] Allport S. (2007). *The Queen of Fats: Why Omega-3s Were Removed from the Western Diet and What We Can Do to Replace Them*.

[30] Yannakopoulos A. L., Tserveni-Gousi A. S. (1986). Quality characteristics of quail eggs. *British Poultry Science*.

[31] Simopoulos A. P. (2002). The importance of the ratio of omega-6/omega-3 essential fatty acids. *Biomedicine & Pharmacotherapy*.

[32] Mensink R. P. (2005). Effects of stearic acid on plasma lipid and lipoproteins in humans. *Lipids*.

[33] Kris-Etherton P. M., Griel A. E., Psota T. L., Gebauer S. K., Zhang J., Etherton T. D. (2005). Dietary stearic acid and risk of cardiovascular disease: intake, sources, digestion, and absorption. *Lipids*.

[34] Nestel P., Clifton P., Noakes M. (1994). Effects of increasing dietary palmitoleic acid compared with palmitic and oleic acids on plasma lipids of hypercholesterolemic men. *Journal of Lipid Research*.

[35] French M. A., Sundram K., Clandinin M. T. (2002). Cholesterolaemic effect of palmitic acid in relation to other dietary fatty acids. *Asia Pacific Journal of Clinical Nutrition*.

[36] Chung H.-Y., Rasmussen H. M., Johnson E. J. (2004). Lutein bioavailability is higher from lutein-enriched eggs than from supplements and spinach in men. *Journal of Nutrition*.

[37] Johnson M. L., Dahiya J. P., Olkowski A. A., Classen H. L. (2008). The effect of dietary sinapic acid (4-hydroxy-3, 5-dimethoxy-cinnamic acid) on gastrointestinal tract microbial fermentation, nutrient utilization, and egg quality in laying hens. *Poultry Science*.

[38] Nimalaratne C., Lopes-Lutz D., Schieber A., Wu J. (2011). Free aromatic amino acids in egg yolk show antioxidant properties. *Food Chemistry*.

[39] Chiba L. I. (2014). *Poultry Nutrition and Feeding*.

[40] Schimmer B. P., Parker K. L. (2006). Adrenocartical steroids and their synthetic analogues. *The Pharmatcological Basis of Therapeutics*.

